# Targeted proteomics addresses selectivity and complexity of protein degradation by autophagy

**DOI:** 10.1080/15548627.2024.2396792

**Published:** 2024-09-08

**Authors:** Alexandre Leytens, Rocío Benítez-Fernández, Carlos Jiménez-García, Carole Roubaty, Michael Stumpe, Patricia Boya, Jörn Dengjel

**Affiliations:** a Department of Biology, University of Fribourg, Fribourg, Switzerland; b Department of Neuroscience and Movement Science, University of Fribourg, Fribourg, Switzerland

**Keywords:** ER-phagy, mass spectrometry, mitophagy, parallel reaction monitoring, reticulophagy, selective autophagy receptors

## Abstract

Macroautophagy/autophagy is a constitutively active catabolic lysosomal degradation pathway, often found dysregulated in human diseases. It is often considered to act in a cytoprotective manner and is commonly upregulated in cells undergoing stress. Its initiation is regulated at the protein level and does not require *de novo* protein synthesis. Historically, autophagy has been regarded as nonselective; however, it is now clear that different stimuli can lead to the selective degradation of cellular components via selective autophagy receptors (SARs). Due to its selective nature and the existence of multiple degradation pathways potentially acting in concert, monitoring of autophagy flux, *i.e*. selective autophagy-dependent protein degradation, should address this complexity. Here, we introduce a targeted proteomics approach monitoring abundance changes of 37 autophagy-related proteins covering process-relevant proteins such as the initiation complex and the Atg8-family protein lipidation machinery, as well as most known SARs. We show that proteins involved in autophagosome biogenesis are upregulated and spared from degradation under autophagy-inducing conditions in contrast to SARs, in a cell-line dependent manner. Classical bulk stimuli such as nutrient starvation mainly induce degradation of ubiquitin-dependent soluble SARs and not of ubiquitin-independent, membrane-bound SARs. In contrast, treatment with the iron chelator deferiprone leads to the degradation of ubiquitin-dependent and -independent SARs linked to mitophagy and reticulophagy/ER-phagy. Our approach is automatable and supports large-scale screening assays paving the way to (pre)clinical applications and monitoring of specific autophagy flux.

**Abbreviation:** AMBRA1: autophagy and beclin 1 regulator 1; ATG: autophagy related; BafA1: bafilomycin A_1_; BNIP1: BCL2 interacting protein 1; BNIP3: BCL2 interacting protein 3; BNIP3L/NIX: BCL2 interacting protein 3-like; CALCOCO2/NDP52: calcium binding and coiled-coil domain 2; CCPG1: cell cycle progression 1; CV: coefficients of variations; CCCP: carbonyl cyanide m-chlorophenyl hydrazone; DFP: deferiprone; ER: endoplasmic reticulum; FKBP8: FKBP prolyl isomerase 8; GABARAPL: GABA type A receptor associated protein like; LC: liquid chromatography; LOD: limit of detection; LOQ: limit of quantification; MAP1LC3: microtubule associated protein 1 light chain 3; MS: mass spectrometry; NCOA4: nuclear receptor coactivator 4; NBR1: NBR1 autophagy cargo receptor; NUFIP1: nuclear FMR1 interacting protein 1; OPTN: optineurin; PHB2: prohibitin 2; PNPLA2/ATGL: patatin like phospholipase domain containing 2; POI: protein of interest; PTM: posttranslational modification; PRM: parallel reaction monitoring; RB1CC1/FIP200: RB1 inducible coiled-coil 1; RETREG1/FAM134B: reticulophagy regulator 1; RPS6KB1: ribosomal protein S6 kinase B1; RTN3: reticulon 3; SARs: selective autophagy receptors; SQSTM1/p62: sequestosome 1; STBD1: starch binding domain 1; TAX1BP1: Tax1 binding protein 1; TFEB: transcription factor EB; TNIP1: TNFAIP3 interacting protein 1; TOLLIP: toll interacting protein; ULK1: unc-51 like autophagy activating kinase 1; WBP2: WW domain binding protein 2; WDFY3/Alfy: WD repeat and FYVE domain containing 3; WIPI2: WD repeat domain, phosphoinositide interacting 2.

## Introduction

Eukaryotic cells maintain homeostasis and remove damaged or superfluous cellular components through a lysosomal degradation pathway called macroautophagy (hereafter referred to as autophagy). This highly conserved catabolic pathway occurs at basal levels but is enhanced by a variety of stress signals, such as nutrient starvation or organelle damage [1]. The process starts with the *de-novo* formation of a double-membrane organelle called the phagophore from membranes being recruited from various sources, the endoplasmic reticulum (ER) likely being the principal donor [2]. This growing cup-shaped membrane engulfs parts of the cytoplasm and closes to form a double-membrane vesicle termed an autophagosome. The autophagosome can fuse with other vesicles from endocytic pathways forming an amphisome and finishes by fusing with the lysosome/vacuole. Lysosomal fusion exposes autophagosomal cargo including the inner membrane to acidic hydrolases and enables the degradation of its content. The generated building blocks are recycled and transported back to the cytosol by lysosomal permeases to generate energy or fuel anabolic pathways.

While basal autophagy is often considered a “bulk”, *i.e*. a nonselective process recycling cellular components in a random manner, autophagy can also be selective. Well studied cases of selective autophagy include the targeted removal of damaged organelles such as the mitochondrion or ER (termed mitophagy and reticulophagy/ER-phagy, respectively) as well as of protein aggregates (aggrephagy). This particular selectivity is enabled by a set of proteins called selective autophagy receptors (SARs) [3], bridging the autophagy cargo to proteins of the Atg8 family, which are lipidated proteins coating the phagophore membrane and functioning as docking sites [4].

Numerous methods and protocols have been described for the study of protein turnover by autophagy, several of them relying on immunodetection or fluorescent microscopy of single proteins, which are often ectopically expressed as tagged variants facilitating downstream analyses [5]. While these methods are well established, they often require a significant protein amount or protein tagging, making them unsuitable for some sample types, *e.g*., primary cells being difficult to transfect, and difficult to adapt for large-scale screening approaches. Additionally, most of these methods infer the activity of the whole pathway based on the quantification of single markers. Whereas this might be relevant for the analysis of basal, *i.e*. nonselective, autophagy flux, such approaches likely fail to capture the complexity of stress-induced autophagy in which different cargoes might get degraded at different rates and through different sub-pathways [6]. To address the complexity of autophagy regulation and activity in an unbiased manner, approaches monitoring expression of multiple genes by RNA-seq have been developed that are adaptable for higher sample throughput and might also be used in prognostic/diagnostic settings [7]. However, to infer autophagy activity based on changes in gene transcription biological samples have to be treated for several hours, which is in contrast to the rapid cellular response to stress conditions. Autophagy signaling is activated within minutes [8], initial autophagosome biogenesis does not require *de novo* protein synthesis [9], and in mammalian cells autophagosomes and autophagy-dependent protein degradation can be detected as early as 30 min post stimulus [10]. Additionally, while transcriptomic approaches are well suited to investigate future adaptations of the cells to a given stimulus, they cannot answer questions about protein degradation itself. To address this gap in analytical approaches, we developed an assay relying on detecting changes in protein abundances, which is compatible with high sample throughput, and which monitors abundance changes of multiple endogenous proteins reflecting the complexity and multitude of autophagy subtypes.

We turned to targeted proteomics, which are mass spectrometry (MS)-based analyses enabling the accurate quantification of a predefined number of given peptides potentially encompassing numerous target proteins [11], supporting absolute protein quantification [12], and being highly sensitive with limits of detection in the Attomole range. Mass spectrometers operated in targeted mode focus on a pre-established set of mass-to-charge ratios corresponding to peptides-of-interest. By ignoring other peptides eluting from the coupled liquid chromatography (LC) column, targeted proteomics greatly enhances the sensitivity for its targets. Combining this approach with labeled spiked-in synthetic peptides further enables confident identification of the targets, robust normalization across samples, and absolute quantification, depending on peptide purity [13]. Here, we describe a targeted proteomics approach based on parallel reaction monitoring (PRM) [14], allowing to accurately quantify tryptic peptides derived from human autophagy-relevant proteins in single MS measurements. In combination with a simple in-solution digestion protocol that can be performed in a (semi)automated manner the assay supports screening approaches. We monitor proteins involved in the autophagic responses to amino acid and glucose starvation, as well as to deferiprone (DFP) treatment, a potent inducer of mitophagy [15], covering autophagy initiation, autophagosome biogenesis and turnover, as well as SARs enabling an accurate assessment of the activity and selectivity of the entire process.

## Results

### Target selection and peptide characterization

**Figure 1.**
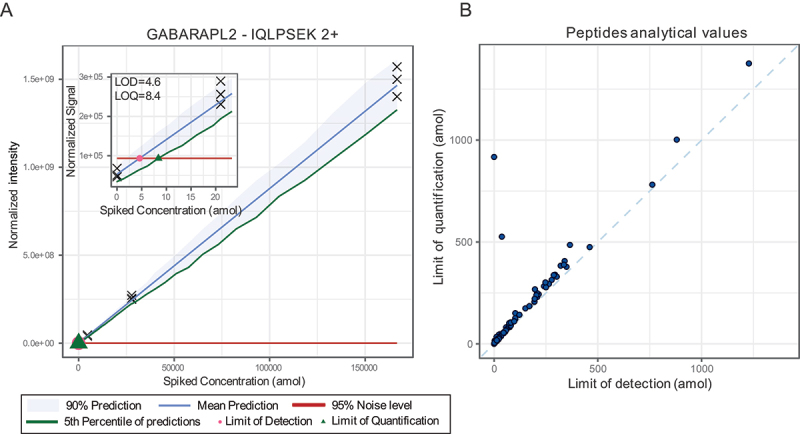
Peptide responses and quantitative accuracy. (A) Example calibration curve of the GABARAPL2 peptide IQLPSEK which was detected as doubly charged precursor (2+) and used to monitor GABARAPL2 protein abundance. Inlay is a zoom of the low concentration range. Black crosses indicate measured data points. (B) Distribution of limits of detection and quantification of the 94 tryptic peptides used to monitor autophagy.

To cover bulk and selective autophagy subtypes by targeted proteomics, we screened the respective literature for relevant proteins [1,4,7,16]. In addition, we included selected, autophagy-relevant proteins from ongoing research projects in our groups [17], which led to a list of 41 proteins-of-interest (POIs) (Table 1). MS-compatible peptides of POIs for targeted proteomics are commonly generated by proteolytic digestion using the endoprotease trypsin. Not all tryptic peptides from a given source protein are suited for targeted MS as some sequences might not fit the requirements of LC (highly hydrophilic or hydrophobic peptides), chemical stability (*e.g*., post-lysis methionine oxidation), or distribution of trypsin cleavage sites (lysine or arginine positions in the sequence prevent digestion into suitable peptides yielding either too large or too small peptides). Therefore, we only considered peptide sequences between 7 and 25 amino acids in length. Due to the sensitivity to oxidations, methionine-containing sequences were excluded. Unfortunately, these restrictions led to the exclusion of some important protein candidates. As an example, human Atg8-family proteins are very short and only generate a limited number of tryptic peptides. Among this small set, some peptides had to be removed as their sequences did not match our quality criteria. As a result, only three peptides from GABARAPL1 and GABARAPL2 were further considered and no LC3-derived peptides (MAP1LC3A, MAP1LC3B, MAP1LC3B2, MAP1LC3C) made it to the final list. As a next step, shortlisted peptides were screened on their detection in previous MS experiments in publicly available MS datasets from PeptideAtlas as well as by our group [41,42]. This led to a selection of 114 peptides sequences from the 41 POIs. These were synthesized following the AQUA principle using isotopically labeled arginine or lysine variants [43], supporting their use as synthetic spike-ins to aid identifications of endogenous peptides. To assess the usability of these peptides, we first determined their linear concentration range, *i.e*., the range in which changes in quantities generate a proportional change in the recorded MS signal (Figure 1).

**Table 1.** Target proteins monitored by PRM.

Protein description	UniProt accession	Gene name	Details	Reference
Autophagy and beclin 1 regulator 1	Q9C0C7	*AMBRA1*	Mitophagy receptor	[18]
Autophagy related 12	O94817	*ATG12*	Lipidation machinery	[19]
Autophagy related 13	O75143	*ATG13*	Initiation	[1]
Autophagy related 2A	Q2TAZ0	*ATG2A*	Lipidation machinery	[19]
Autophagy related 3	Q9NT62	*ATG3*	Lipidation machinery	[19]
Autophagy related 4A cysteine peptidase	Q8WYN0	*ATG4A*	Lipidation machinery	[19]
Autophagy related 4B cysteine peptidase	Q9Y4P1	*ATG4B*	Lipidation machinery	[19]
Autophagy related 5	Q9H1Y0	*ATG5*	Lipidation machinery	[19]
Autophagy related 7	O95352	*ATG7*	Lipidation machinery	[19]
Autophagy related 9A	Q7Z3C6	*ATG9A*	Autophagosome biogenesis	[1]
BCL2 interacting protein 1	Q12981	*BNIP1*	Autophagosome biogenesis	[20]
BCL2 interacting protein 3	Q12983	*BNIP3*	Mitophagy receptor	[21]
BCL2 interacting protein 3 like	O60238	*BNIP3L*	Mitophagy receptor, pexophagy receptor	[22]
Calcium binding and coiled-coil domain 2	Q13137	*CALCOCO2*	Ubiquitylated substrate receptor	[23]
Cell cycle progression 1	Q9ULG6	*CCPG1*	Reticulophagy receptor	[24]
FKBP prolyl isomerase 8	Q14318	*FKBP8*	Mitophagy receptor	[25]
GABA type A receptor-associated protein	Q9GJW7	*GABARAP*	Mammalian Atg8 protein	[19]
GABA type A receptor associated protein like 1	Q9H0R8	*GABARAPL1*	Mammalian Atg8 protein	[19]
GABA type A receptor associated protein like 2	P60520	*GABARAPL2*	Mammalian Atg8 protein	[19]
Microtubule associated protein 1 light chain 3 alpha	Q9H492	*MAP1LC3A*	Mammalian Atg8 protein	[19]
Microtubule associated protein 1 light chain 3 gamma	Q9BXW4	*MAP1LC3C*	Mammalian Atg8 protein	[19]
NBR1 autophagy cargo receptor	Q14596	*NBR1*	Ubiquitinated substrate receptor, pexophagy receptor	[26]
Nuclear receptor coactivator 4	Q13772	*NCOA4*	Ferritinophagy receptor	[27]
Nuclear FMR1 interacting protein 1	Q9UHK0	*NUFIP1*	Proposed ribophagy receptor	[28]
Optineurin	Q96CV9	*OPTN*	Ubiquitinated substrate receptor	[29]
Prohibitin 2	Q99623	*PHB2*	Mitophagy receptor	[30]
Patatin like phospholipase domain containing 2	Q96AD5	*PNPLA2*	Lipophagy receptor	[31]
RB1 inducible coiled-coil 1	Q8TDY2	*RB1CC1*	Autophagy initiation	[1]
Reticulophagy regulator 1	Q9H6L5	*RETREG1/FAM134B*	Reticulophagy receptor	[32]
Ribosomal protein S6 kinase B1	P23443	*RPS6KB1*	Signaling	[33]
Reticulon 3	O95197	*RTN3*	Reticulophagy receptor	[34]
Sequestosome 1	Q13501	*SQSTM1*	Ubiquitylated substrate receptor	[35]
Starch binding domain 1	O95210	*STBD1*	Glycophagy receptor	[36]
Tax1 binding protein 1	Q86VP1	*TAX1BP1*	Ubiquitinated substrate receptor	[37]
Transcription factor EB	P19484	*TFEB*	Lysosomal biogenesis	[1]
TNFAIP3 interacting protein 1	Q15025	*TNIP1*	Autophagy receptor	[17]
Toll interacting protein	Q9H0E2	*TOLLIP*	Ubiquitinated substrate receptor	[38]
Unc-51 like autophagy activating kinase 1	O75385	*ULK1*	Signaling, initiation	[1]
WW domain binding protein 2	Q969T9	*WBP2*	Autophagosome biogenesis	
WD repeat and FYVE domain containing 3	Q8IZQ1	*WDFY3/Alfy*	Ubiquitylated substrate receptor	[39,40]
WD repeat domain, phosphoinositide-interactingprotein 2	Q9Y4P8	*WIPI2*	Autophagosome biogenesis	[1]

As with all analytical techniques, quantitative accuracy can only be achieved within a certain analyte concentration range. Quantitative accuracy of targeted proteomics assays is achieved by determining the linear range of detection, the limit of detection (LOD) and the limit of quantification (LOQ) of respective analytes, *i.e*., the analyte concentrations at which the method generates a signal clearly separated from the noise and a reproducible signal, respectively. These values are peptide-specific and depend in part on the way peptides ionize; hence, they must be determined experimentally for all the target analytes by measuring calibration curves using concentration gradients. For this we followed the envisioned workflow of the assay with the exception that synthetic peptides were spiked after protein digestion (see Materials and Methods for details). Briefly, A549 human lung carcinoma cells were harvested, whole cell lysate was generated, and proteins digested overnight using trypsin. To different aliquots of the same starting material, *i.e*., 30 µg of trypsin-digested proteins of whole cell lysate, different amounts of synthetic peptides were spiked, and samples were analyzed by quantitative LC-MS/MS (see Methods for details).

**Figure 2.**
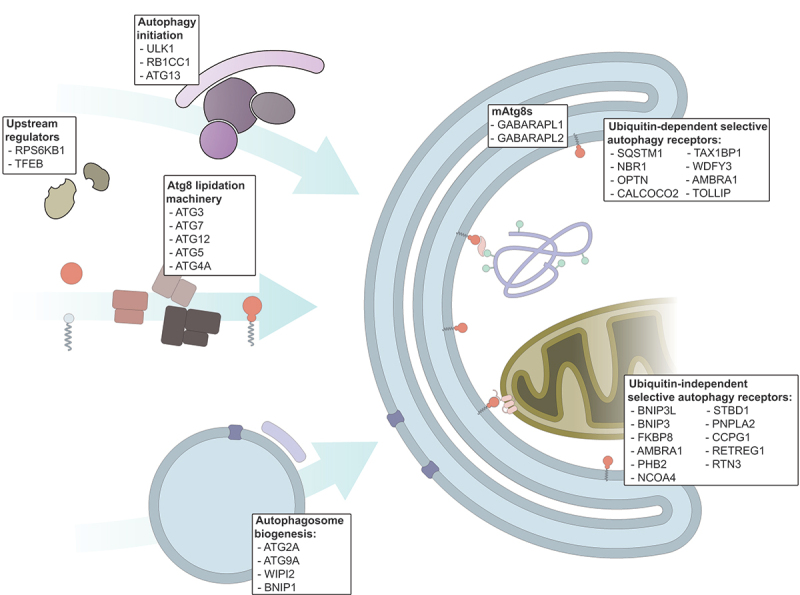
Selected protein targets. Schematic representation of autophagy initiation, highlighting the selected target proteins and their function in autophagy.

The calibration curves for the targeted peptides were measured in three replicates across 7 different concentrations from 6-fold serial dilutions (0.021 fmol to 1`000 fmol on LC column) and three blank samples. The peptide amounts mentioned throughout this manuscript were calculated based on product weight used for preparing the solutions. Peptide purity was however not 100% and therefore actual peptide quantities are likely to be lower. As we do not aim for absolute quantification and as these values are only used comparatively between measurements, we provide quantities as indicative values. To only consider the technical variations introduced by the LC-MS/MS setup and not the sample preparation protocol, the triplicate measurements were performed using the same samples. Each replicate of the calibration curve was run in increasing concentration order and blank samples were ran before starting the next curve to limit carry over effects. Only peptide precursors for which a minimum of three fragment ions were detected were used for further analyses. Thus, per peptide a minimum of three LC-MS/MS elution profiles were used for quantification. For most measured peptides, the highest measured concentration (1 pmol on LC column), caused a loss of signal linearity and/or produced tails in the chromatograms. For this reason, this concentration was not considered for the fitting of the calibration curve. The remaining points were used to fit a calibration curve using the MSStats LOB/LOD package [44]. Briefly, this method enables to fit a nonlinear regression model to the intensity-concentration response curve. LOD and LOQ can then be derived from this model. LODs were defined as the concentrations corresponding to the intersection of the upper bound of the intensity prediction interval for the blank samples (corresponding to the noise level) and the mean predicted intensity of spiked amounts. In other words, the LOD of a peptide was set as the smallest concentration for which a signal can be separated from the noise defined by the blank samples. LOQs were set as the smallest concentrations corresponding to the intersection between the upper bound of the predicted noise intensity and the lower bound of the confidence interval of predicted intensities for that concentration (see Figure 1 as an example). Thus, the more reproducible the measurement, the lower the LOQ as the confidence interval of the predicted response is narrower allowing a clear discrimination from chemical noise. Out of the 114 considered peptides, 20 could either not be reproducibly detected or accurately quantified and were removed from further considerations, leading to a final list of 94 peptides from 37 POIs with quantified LODs and LOQs (Figure 2, Table S1). The chosen targets include proteins relevant to autophagy regulation (RPS6KB1 and TFEB), the initiation complex (ULK1, RB1CC1/FIP200, ATG13), autophagosome biogenesis (ATG2A, ATG9A, WIPI2, BNIP1, WBP2), the Atg8-family lipidation machinery (ATG3, ATG7, ATG12, ATG5, ATG4A) and mammalian Atg8-family proteins (GABARAPL1, GABARAPL2), as well as SARs (SQSTM1/p62, NBR1, OPTN, CALCOCO2/NDP52, TAX1BP1, WDFY3/Alfy, CCPG1, RETREG1/FAM134B, RTN3, BNIP3L/NIX, TOLLIP, FKBP8, BNIP3, AMBRA1, PHB2, NCOA4, STBD1, PNPLA2/ATGL, NUFIP1, TNIP1) (Figure 2).

We did not consider modified peptides in the current variant of the assay; however, as several of the monitored peptides have been reported to potentially carry PTMs (Table S2), quantification accuracy of respective source proteins might be affected. To test if PTMs of peptides potentially influence protein quantification accuracy, we checked the pairwise correlations of peptides from the same source proteins and found in general a good correlation, with a median correlation coefficient > 0.82 in all experiments (Figure S1). The few peptide pairs which showed a correlation of less than 0.5 were in general linked to very low abundant signals, indicating that the low correlation is likely due to technical/biological noise. As PTMs are commonly sub-stoichiometric and only a small percentage of a given peptide might actually carry the respective PTM, and as we did not inhibit PTM removing enzymes such as phosphatases and deubiquitinases, we conclude that modifications of the used peptides if at all play only a minor influence and should not affect quantification accuracy of listed proteins.

### Robustness of the assay compared to standard MS workflows and in a biological context

**Figure 3.**
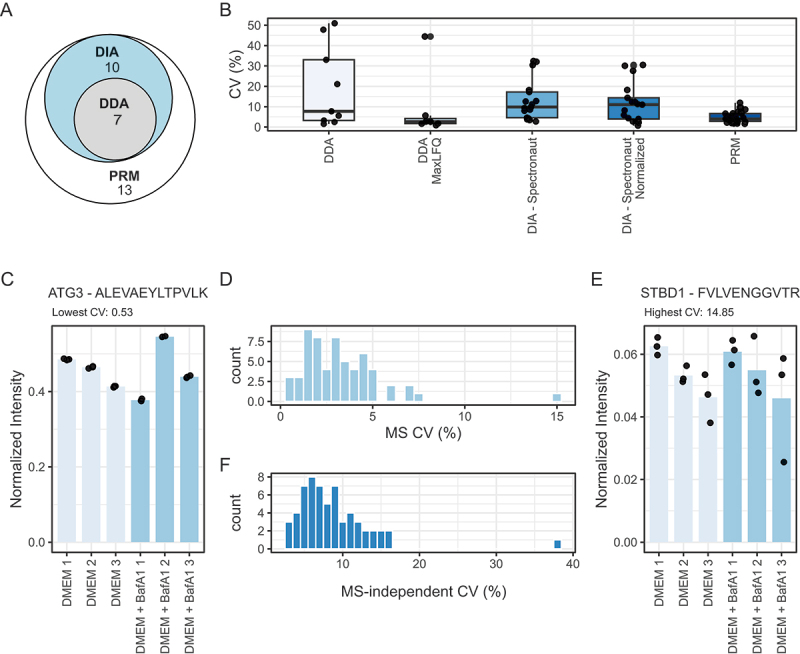
Methods comparisons and measurements of CVs. (A) Euler plot of target proteins quantified in a minimum of one condition in DIA (Spectronaut normalization), DDA (MaxLFQ) and PRM. (B) Average coefficients of variations at the protein level across HBSS and HBSS + BafA1 treatments using different quantification methods. Single values are represented by black dots. (C–E) Average peptides coefficients of variations distribution within biological replicates (d). Detailed data from both lowest and highest CV producing peptides derived from (c) ATG3 and (e) STBD1, respectively, are shown as examples. (F) peptide CVs across biological replicates, representing biological variation and variation introduced by sample preparation.

Amino acid starvation is a strong and commonly used autophagy inducing stimulus. Coupling it to bafilomycin A_1_ (BafA1) treatment, which inhibits V-type proton-ATPases blocking lysosomal degradation [5], enables to measure the accumulation of autophagosomal cargo that would be degraded in the absence of BafA1 treatment. To assess the reproducibility of our approach and compare it to standard shotgun proteomics approaches, A549 cells were starved for amino acids in Hanks’ Balanced Salt Solution (HBSS) with and without BafA1 for 2 h, samples processed as outlined above, and resulting peptide mixtures were measured on the same LC-MS/MS system comparing PRM-based targeted proteomics to Data-Dependent Acquisition (DDA)-, and Data-Independent Acquisition (DIA)-based discovery proteomics (*n* = 3 technical replicates per treatment). Out of the 37 protein targets of the assay, 30 could be identified and quantified with a signal above the LOQ in a minimum of one sample using PRM. Standard DIA measurements identified 17 of these proteins and DDA identified 7 (Figure 3A). When it comes to quantification, DDA measurements resulted in large coefficients of variation (CVs). Normalized DDA measurements using the MaxLFQ algorithm [45] performed surprisingly well with an average CV of 8.5%; however, only 7 proteins were retained and depending on the protein the CV might be significantly higher (max. value of 44%). Not surprisingly, targeted proteomics developed with specific targets in mind outperformed the other approaches when measuring those targets. In terms of reproducibility, PRM measurements produced an average CV across conditions of 4.8% with most proteins being below 10% (Figure 3B).

We then tested the reproducibility of the PRM assay on a larger sample number, using cells harvested in growth conditions (DMEM) in the absence and presence of BafA1 for 2 h. Biological triplicates were prepared for these conditions and all of them were measured 3 times to clearly separate the noise originating from biological differences and sample preparation from the purely technical MS-related noise. For these quantifications, 60 peptides which were identified without interferences in all samples, and which gave rise to a signal above the LOQ were considered for CV calculations. CVs calculated for all technical triplicates and averaged across samples ranged from 0.5% (peptide ATG3[12-24]: ALEVAEYLTPVLK) to 14.8% (peptide STBD1[323-333] FVLVENGGVTR) with a majority below 5% showing high technical reproducibility of the assay with variations lower or in the range of the variation observed across biological replicates (Figure 3C–F).

### Assessment of stimulus-dependent protein degradation by autophagy

Having established the technical principles for our assay, we next asked if we could identify differences in autophagy-dependent protein regulation comparing 2 h amino acid starvation and 2 h glucose starvation to growth conditions in complete media (DMEM). These treatments were coupled to BafA1 treatments for 2 h to study the autophagy flux in all three conditions. Some conditions like amino-acid starvation (HBSS) considerably reduced protein levels of autophagy cargoes making it difficult to detect and/or quantify these peptides. To calculate protein quantities, we used the following strategy: if a set of peptides was identified in all samples and resulted in a signal above the LOQ, only this set was used for inferring the protein amount. If no valid peptide was found across the whole experiment the protein was considered missing and not quantified. Lastly, if a protein was identified with a given valid peptide set in some samples but none of these peptides were valid in other samples, the protein abundance was imputed with the same standard deviation as the one observed for measured samples and a downshifted mean of 20%. We regard this as a rather conservative estimation of protein abundance in the case of missing values and it can easily be adjusted based on the biological setting of the experiment. This strategy led to the quantification of 34 proteins out of the list of 37 POIs with peptides above the LOQ (Table S3).

**Figure 4.**
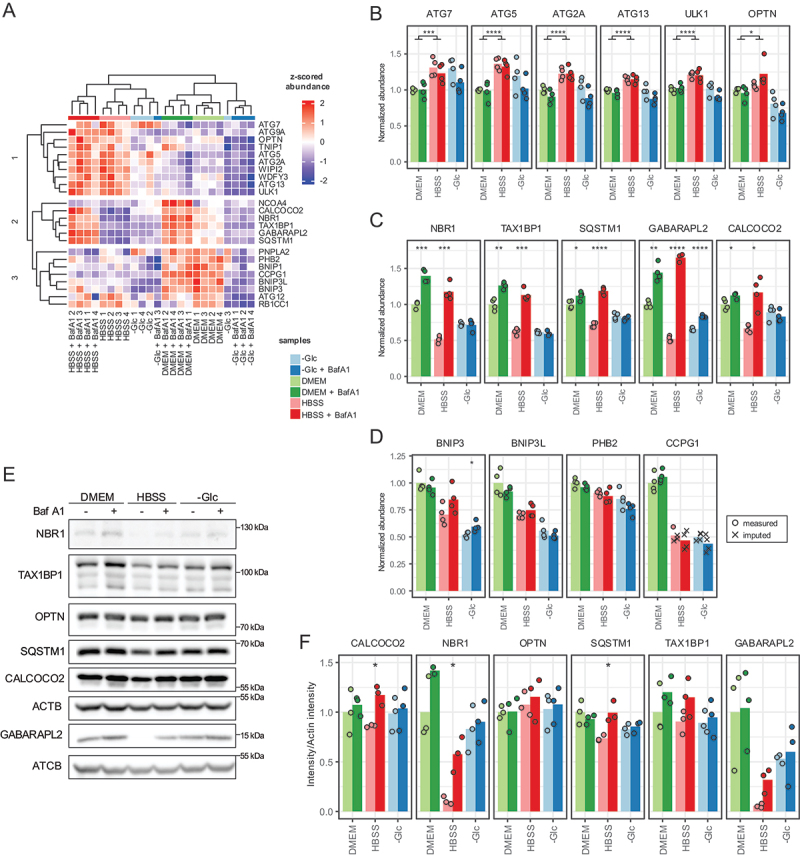
Regulation of protein abundances by autophagy upon different starvation stimuli in A549 cells. (A) Heatmap of a hierarchical cluster analysis summarizing the behavior of regulated proteins reaching a p-value <0.01 in an ANOVA test across all conditions (2 h treatments). (B–D) Example proteins for the clusters 1, 2 and 3 from panel a, respectively. Basal conditions (DMEM) were set as 1 for reference. (E) Western blot analyses of some of the targets of the proteomics assay. Shown is one representative of *n* = 3 biological replicates. Actin was used for normalization. (F) Quantification from the western blots shown in (e). Asterisks represent significance level of a two-side unpaired Student t-test between conditions in (b) and within a condition ± BafA1 treatment in (c,d, f). *=p < 0.05, **=p < 0.01, ***=p < 0.001, ****=p < 0.0001, ns=not significant. Bars highlight average values and dots single experiments.

To identify potential patterns of differential regulation of these proteins across the tested conditions, we filtered the results for proteins showing a significant difference in abundance between treatments (p-value <0.01 in one-way ANOVA). This filtering yielded a total of 24 proteins. Upon clustering of the results, experimental conditions could be clearly separated, and three major clusters emerged (Figure 4A). Cluster 1 is mostly comprised of components of the core autophagy machinery (Figure 4A,B). These proteins do not show degradation patterns in any of the tested conditions but seem to accumulate under amino-acid starvation. The levels of these proteins consistently and significantly increase by about 20% once cells are starved for amino acids, regardless of lysosomal activity (Figure 4B, *p* < 0.05, T test).

Cluster 2 constitutes of ubiquitin-dependent, soluble SARs and the ATG8 homolog GABARAPL2 (Figure 4A,C). SARs like SQSTM1 are being turned over to some extent under basal conditions and amino-acid starvation drastically increases their flux, corroborating their central role in adaptation to amino acid starvation. In cluster 3 membrane-bound, ubiquitin-independent autophagy receptors BNIP3, BNIP3L, PHB2 and CCPG1 are not being degraded within lysosomes under any conditions but interestingly seem to be at their most abundant levels under growth conditions (Figure 4A,D). The mechanism leading to their decrease under stress conditions is not known. For some proteins, the trends observed by targeted proteomics were also observed by immunoblotting, although the noise levels were higher (Figure 4E,F). Interestingly, glucose starvation decreases the overall level of SARs compared to growth conditions. This decrease is independent of lysosomal activity as BafA1 treatment has no effect (Figure 4C). The block of turnover is also reflected in the poor clustering of the samples starved for glucose with and without BafA1, as this drug should underscore the autophagy-related cargo degradation. The small but significant stabilization of GABARAPL2 by BafA1 indicates that lysosomes are principally still active, questioning the role of the monitored proteins in autophagy-dependent adaptation to glucose starvation.

**Figure 5.**
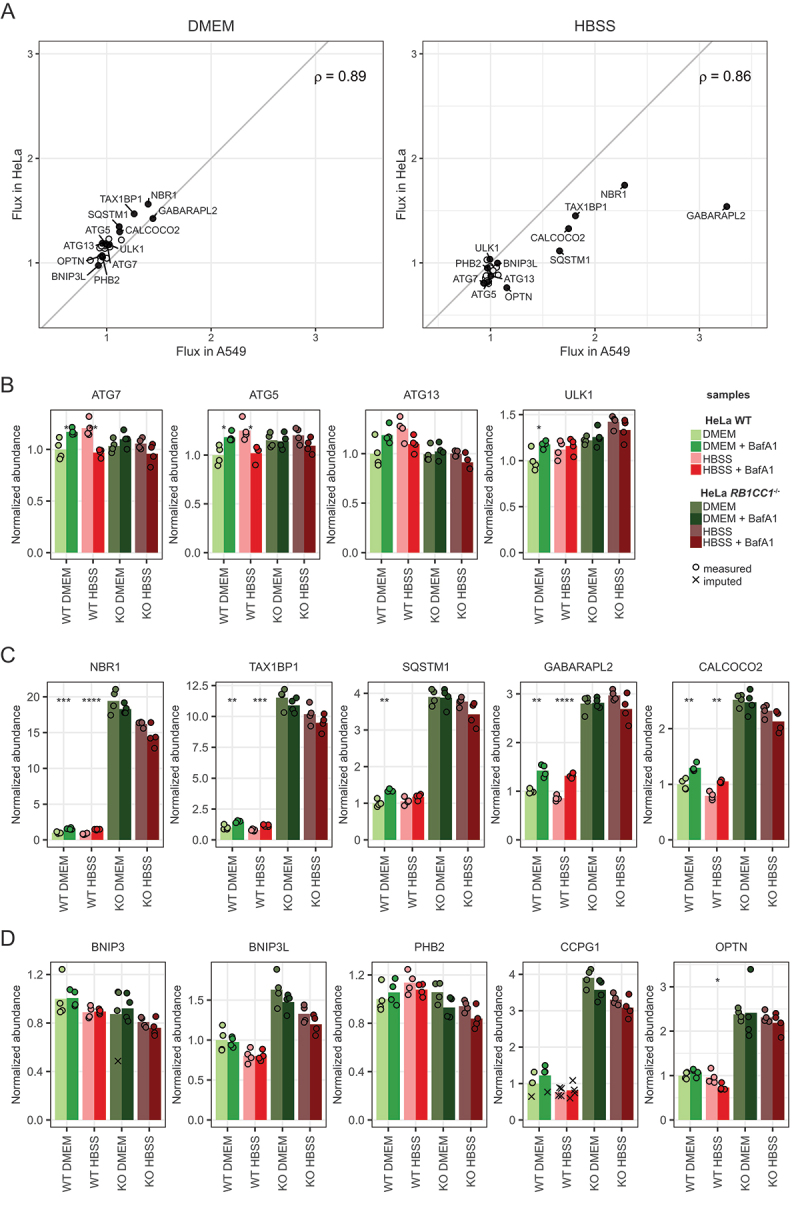
Regulation of protein abundances by autophagy upon different starvation stimuli in HeLa cells. (A) Protein flux in two different cell lines. Flux is defined as the mean of a condition treated with BafA1 divided by the mean of the same condition without BafA1 for all considered proteins. Proteins represented in panels (b-d) are annotated. (B–D) Bar plots representing the changes in protein abundances across conditions. Basal conditions (DMEM, wild-type cells) were set as 1 for reference. Asterisks represent significance level of a two-sided unpaired Student t-test within a condition ± BafA1 treatment. *=p < 0.05, **=p < 0.01, ***=p < 0.001, ****=p < 0.0001.

Using the same heavy peptide standards, we investigated whether these results were cell line-specific, or transferable to other cell lines by analyzing HeLa cells. As the effect of glucose starvation seemed limited, we focused on growth conditions and 2 h of amino-acid starvation, both in the absence and presence of BafA1. In addition, we included *RB1CC1/FIP200* KO HeLa cells to test whether the observed accumulation of some SARs was indeed macroautophagy dependent (Figure 5, Table S4). Overall, protein flux in A549 and HeLa wild-type cells correlated well, with a Pearson correlation coefficient of 0.89 for growth, and 0.86 for starvation conditions, respectively (Figure 5A). The slight decrease of linear correlation in starvation compared to growth conditions, which is particularly vivid for highlighted SARs and GABARAPL2, might indicate a similar regulation of protein flux in basic autophagy, and cell type-specific changes in stress-induced autophagy. Interestingly, HeLa cells did not exhibit the increase of the core autophagy machinery in starvation conditions in contrast to A549 cells (Figure 5B). In HeLa cells, ATG7, ATG5 and ULK1 seem to be turned over in lysosomes under growth, but not anymore under starvation conditions. Just like in A549 cells, soluble ubiquitin dependent autophagy receptors like SQSTM1, CALCOCO2, NBR1 and TAX1BP1 showed significant autophagy flux under growth condition. All of them had an increased flux upon starvation except for SQSTM1, which seemed rather stable in HeLa cells under these conditions (Figure 5C). Also in HeLa cells, ubiquitin independent SARs did not show any flux under the tested conditions (Figure 5D). In the case of *RBICC1*-KO cells, our method nicely confirmed the absence of signals from peptides derived from RB1CC1 (Figure S2). Additionally, we observed a large accumulation of SARs and GABARAL2 in these cells that were not affected by BafA1 treatment, supporting the interpretation that the observed protein flux in WT cells are indeed autophagy dependent. A strong accumulation could also be observed for SARs for which flux could not be detected in HeLa wild-type cells, like CCPG1, BNIP3L and OPTN. This suggests that their protein levels are at least partially dependent on macro-autophagy and that 2 h of amino acid starvation might be too short to detect significant autophagy-dependent turnover of these proteins.

### Determination of protein regulation during mitophagy

**Figure 6.**
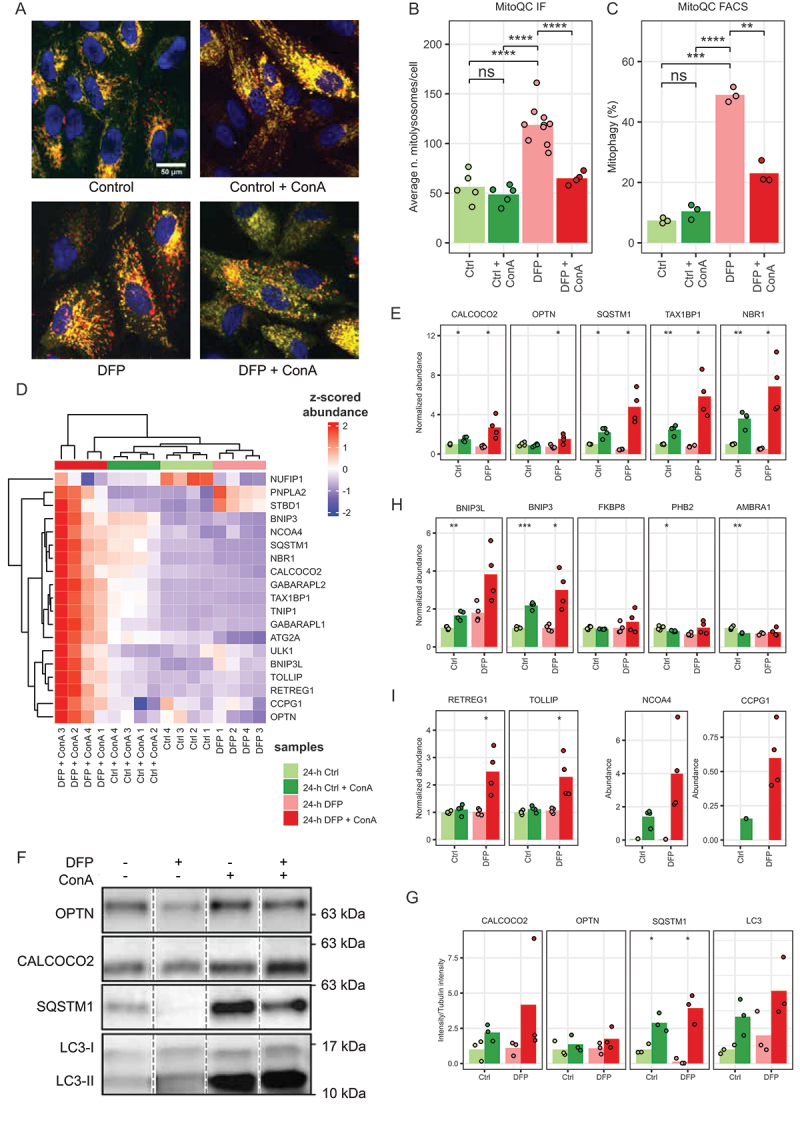
PRM measurements of DFP treated cells. (A) Immuno-fluorescence microscopy of *mito*-qc cells. A representative of *n* = 5-10 biological replicates is shown for a total of 200 cells. (B) Quantification of the *mito*-qc reporter-based experiments highlighted in (a). Black dots represent the number of mitolysosomes per cell. (C) Flow cytometry analysis of *mito*-qc cells. Shown is one representative of *n* = 3 biological replicates. (D) Heatmap summarizing the changes in protein regulation upon mitophagy induction with DFP. Represented proteins reach a p-value <0.01 by ANOVA test. (E) detailed quantification of soluble SQSTM1-like autophagy receptors linked to mitophagy. (F) Western blot analysis of indicated target proteins. Shown is one representative experiment of *n* = 3 biological replicates. Note: dashed lines indicate cropping marks of unrelated samples. All conditions were run on the same gel and blot. (G) quantification of western blots exemplified in (f). (H) Detailed quantification of ubiquitin-independent autophagy receptors involved in mitophagy. (I) Detailed quantification of other autophagy receptors. Only measured datapoints are represented. If not enough datapoints were measured for DMEM condition (reference point), the samples were not normalized to DMEM. *=p < 0.05, **=p < 0.01, ***=p < 0.001, ****=p < 0.0001, ns=not significant, two-sided unpaired Student`s T test. Bars highlight average values and dots single experiments.

After demonstrating that our method detects differences in protein regulation within 2 h of amino acid starvation, we wanted to assess whether we could monitor the flux of specific SARs after inducing selective autophagy. We used ARPE-19 cells, a retinal pigment epithelia cell line, treated with the iron chelator DFP to induce selective BNIP3- and BNIP3L-dependent and PINK1-PRKN/Parkin-independent mitophagy [46]. These cells expressed the *mito*-QC reporter, a FIS1 mCherry-GFP fusion protein enabling to monitor mitochondrial turnover in the lysosome using fluorescence-based approaches [46]. Briefly, this fusion protein emits both red and green fluorescence signals until it is brought to the lysosome, where the acidic conditions quench GFP resulting in a red-only signal that can be detected by flow cytometry or microscopy. We treated cells with DFP for 24 h with and without concanamycin A (ConA), another V-type H^+^-ATPase inhibitor used to monitor accumulation of autophagosomal cargoes [5]. After 24 h of DFP treatment, we observed a significant increase in red mito-lysosomes compared to non-treated control cells by IF microscopy (Figure 6A,B). GFP-quenching in lysosomes could be blocked by ConA. Also, flow cytometry analyses confirmed these results (Figure 6C).

Having established that the *mito*-QC reporter cells behave as anticipated, we performed replicate experiments and subjected them to targeted proteomics using the same heavy peptide standards as outlined above. Protein abundance data were again clustered to gain insights into differential protein regulation. Soluble SARs like SQSTM1, NBR1, CALCOCO2, TAX1BP1, and OPTN, the latter three being also linked to PINK1-PRKN-dependent mitophagy [47,48], show an increased turnover after DFP treatment (Figure 6D,E, Table S5). These trends were also observed by immunoblotting, although the data was noisier (Figure 6F,G). In contrast to the starvation experiments summarized in Figures 4 and 5 , DFP treatment led also to a turnover of membrane-bound SARs: the mitophagy receptors BNIP3 [49] and BNIP3L, which is described as a major mitophagy receptor involved in DFP-dependent mitophagy [50], showed low turnover under growth condition but strongly accumulated in DFP treatment and blocked lysosomal degradation (Figure 6D,H). AMBRA1, FKBP8 and PHB2 which have also all been linked to mitophagy did not respond to the outlined treatments [25,30,51,52]. Surprisingly, other receptors also seemed to strongly respond to DFP treatment, including TOLLIP (a ubiquitin receptor) [38], NCOA4 (a ferritinophagy receptor) [27], and CCPG1 and RETREG1/FAM134B (two reticulophagy receptors) [24,32]. The parallel accumulation of RETREG1/FAM134B and CCPG1 suggests an increased reticulophagy upon DFP treatment which, to the best of our knowledge has not been described yet, despite DFP being a commonly used iron chelator. This emphasizes the need to comprehensively monitor autophagy receptors to fully grasp cellular responses to stresses.

## Discussion

Autophagy research is a very active field of study, and the recent discovery of SARs will undoubtably change our understanding of its physiological relevance. To deepen our understanding of autophagy, these more recent advances need to be considered. Currently, most assays used to study autophagy activity rely on the analyses of single proteins. However, a multitude of selective autophagy subtypes has been discovered [1,4], which may function in concert. When it is not known which organelle is affected by a given stimulus or pharmacological intervention, it is not trivial to decide which proteins should be analyzed to study the involvement and effects of autophagy. Even if a specific autophagy subtype such as mitophagy is analyzed, the usage of different reporter proteins may yield different results concerning autophagy activity [53]. Here, we describe a highly accurate and reproducible, automatable targeted proteomics assay that can be used with low protein amounts in high throughput to screen the autophagic response of cells or tissue specimens in culture. Using this assay, up to 37 autophagy-relevant proteins were identified and quantified, with a focus on SARs.

We would like to point out that the current peptide list is not comprehensive. Several hundred peptides can be accurately monitored by current targeted proteomics approaches [12]. In addition, this method does not require genetic manipulations making it suitable for a wide range of samples. While the targets presented here are specific for human samples, the basic principles of this method can be adapted to virtually any organism of interest. Since the start of this project, additional SARs such as the golgiphagy receptors YIPF3 and YIPF4, or the lipophagy receptor SPART (spartin) have been identified [54,55], and we plan to include additional peptides to get an even more detailed picture of autophagy regulation. Also, usage of additional proteases, such as ProAlanase [17], next to trypsin yields a better sequence coverage, supporting the monitoring of additional target proteins such as the Atg8-family variants that were missed in the current variant of the assay. Importantly, targeted proteomics can also be used to quantify modified peptides, such as ubiquitinated or phosphorylated peptides [56,57], which will allow to monitor kinase activities and autophagy-relevant signal transduction.

In the current setup, we still rely on the blockage of lysosomal activity to study protein accumulation and to determine autophagy flux. BafA1 or ConA were used for this, which inhibit V-ATPase blocking lysosomal acidification and by this hydrolysis activity [5]. To use compounds like BafA1 or ConA, relevant concentration ranges have to be determined which might vary between cell types [58]. In yeast, a more robust assay is widely used: the GFP-cleavage assay which monitors the accumulation of protease resistant GFP using GFP-fusion reporters [5]. A similar strategy has recently been transferred to mammalian cells monitoring the accumulation of protease-resistant, ligand-bound HaloTag [10]. Whereas this assay does not require pharmacological interference with lysosomal activity, it relies on the ectopic expression of tagged marker proteins. We envision that a combination of both approaches, our targeted proteomics approach for screening relevant conditions and a processing assay using the characterized novel targets will yield the most robust assessment of autophagy selectivity and activity. Also, to screen perturbance of autophagy activity in tissue specimens, monitoring of abundance changes of SARs might be sufficient, which would circumvent the need to block lysosomal degradation and would allow analyses of stored specimens, *e.g*. frozen or formalin-fixed, paraffin-embedded tissues, by the outlined approach. More data are needed to robustly address this possibility.

We could demonstrate the suitability of targeted proteomics to measure mammalian Atg8 orthologs, classical markers of so called “bulk” autophagic activity, which are degraded under basal conditions but whose flux drastically increase upon amino acid starvation. Interestingly, it appeared that several proteins of the core autophagy machinery were more abundant under the same conditions in A549 cells. As the observed significant increase is in the range of 20%, such differences would be difficult to detect by expression proteomics or quantitative western blots, highlighting the strength of our approach. As the increase is observable within 2 h of treatment a posttranscriptional regulation likely contributes to the observed abundance changes. Two alternatives come to our minds: (I) ATG proteins could be turned over by a basal non-lysosomal mechanism, which is blocked under stress conditions, *e.g*. the proteasome, or (II) ATG proteins are actively excluded from lysosomal degradation under stress. Since a fixed amount of proteins per assay is used, it could be that “bulk” autophagy is very much upregulated under amino-acid starvation and this leads to a decrease of the overall protein amount whereas the autophagy machinery remains constant, *i.e*., we do not detect an absolute increase in protein amount but a relative increase in the proportion of autophagic machinery in stressed proteomes. Further experiments are needed to answer these questions. However, a similar increase in core autophagy machinery under amino-acid starvation could not be observed in HeLa cells. This is coherent with the different changes in autophagy flux observed in the two cell lines, and it is very likely that different cell lines respond differently to stresses. This highlights the need to study different types of cells and different stresses to better understand the homeostatic function of autophagy. Another interesting observation is that glucose starvation blocked all autophagy activity in the used experimental setup. Why this is the case and if this is compensated by proteasomal activity or is due to reduced transcription/translation is unclear. As lysosomal degradation is commonly regarded as more energy efficient compared to proteasomal degradation [59,60], a general block in all metabolic activity due to the dependency of cancer cell metabolism on glucose availability seems more likely. Analysis of primary, non-transformed cells may shed more light onto this phenomenon. Finally, we also observed that members of the same protein complex are not necessarily coregulated, *e.g*. whereas ULK1 and ATG13 were upregulated by starvation in A549 cells, this was not the case for RB1CC1. If this indicates that RB1CC1 participates in more than one complex under the tested conditions or functions also as a monomer will also have to be determined in future studies.

We then used the same approach to monitor selective autophagy of mitochondria after iron chelation using DFP. Due to the prolonged timing of this stimulus, we observed more prominent autophagy flux as in the starvation experiments. Under DFP treatment, ubiquitin-dependent SARs like SQSTM1 and mammalian Atg8-family proteins showed clear autophagy-dependent turnover. In contrast to the starvation experiments, the membrane-bound SARs known to orchestrate this stress response, BNIP3 and BNIP3L, were identified as clearly upregulated after iron chelation and with a larger flux than under basal conditions. The comprehensive analysis of multiple SARs revealed also a possible increase in ferritinophagy by turnover of the SAR NCOA4. Indeed, DFP treatment was recently shown to also induce ferritinophagy and pexophagy [50,61]. We also identified a potential upregulation of reticulophagy by turnover of CCPG1 and RETREG1 upon DFP treatment which, as far as we know, has not yet been described. DFP treatment leads to a complex cell response, similar to hypoxia having profound influence on the cellular proteome [62,63]. If the observed cellular response is linked to mitophagy as such or is specific to DFP-treatment is not clear and can be tested *e.g*. by using differentially acting, additional mitophagy inducers such as the uncoupler CCCP [64] or the CUL (cullin)‐RING ligase inhibitor MLN4924 [65]. Interestingly, RETREG1 was initially reported to be responsible for basal ER sheet turnover [32], which we did not observe here and likely points toward cell-type specific regulation of selective autophagy. These data nicely highlight the need for incorporating multiple receptors into screening experiments. Due to the redundancy of the system, it can be difficult to get a clear readout of organelle flux and this approach is best combined with more targeted approaches like the *mito*-QC reporter system, which in our case confirmed the engulfment of mitochondria into lysosomes by DFP treatment.

As most published research focuses on a very narrow subset of receptors, we only have limited understanding about the physiological responses to stress conditions and the interplay of selective autophagic activities. We strongly believe that our approach offers a more detailed picture of the actual response to various stresses within a cell. By automating MS sample processing, the described assay is easily adaptable for high sample throughput [66,67], supporting *e.g*. drug screening approaches to characterize pharmacological autophagy modulators. Due to its accuracy, sensitivity, and throughput, we see the applicability of this assays in both basic/preclinical science to study molecular mechanisms of autophagy as well as in clinical science, be it prognosis or diagnosis of health states. The goal will be to identify robust protein biomarkers whose abundance profiles reflect the autophagy state of a given biological specimen.

## Materials and methods

### Cell culture

A549 lung carcinoma cells, HeLa WT and HeLa *RB1CC1* KO cells [68] were grown in Dulbecco’s Modified Eagle Medium (DMEM; PAN biotech, P04–04510) supplemented with 10% fetal bovine serum (FBS; BioWest, S181B–500) and penicillin-streptomycin (PAN Biotech, P06–07100). Cells (2.2 × 10^6^) were seeded into 10-cm dishes to reach about 80% confluency on the next day. Treatments were performed by washing the plates twice with PBS (PAN Biotech, P04–36500) before changing to the treatment medium for 2 h. Treatment media were Hanks’ Balanced Salt Solution (HBSS; Thermo Fisher 14,025,100) for amino-acid starvation and DMEM glucose-free (-Glc; PAN biotech, P04–01548) supplemented with 10% dialyzed FBS, penicillin-streptomycin and stable glutamine (GlutaMAX; Thermo Fisher 35,050,038). Bafilomycin A_1_ (Santa Cruz Biotechnology, sc-201550A) was used at a final concentration of 2 nM. Cells were washed twice with ice-cold PBS before harvesting by scrapping on ice. Cell pellets were snap frozen in liquid nitrogen.

The ARPE-19 (human retinal pigment epithelial cell line) stably expressing the *mito*-QC reporter cell lines was generated in the laboratory of Dr. Ian Ganley [46]. Cells were grown in Dulbecco’s Modified Eagle Medium low glucose (DMEM; Roth, 9005.1) supplemented with Ham’s Nutrient Mixture F12 (Sigma, N6760) 10% FBS (Panbiotech, P30–3306), glutamine (Roth, 9183.1) and penicillin-streptomycin (Gibco 15,140,122). Cells (500,000) were seeded into 6-well plates to reach about 80% confluency on the next day. Cells were then treated with the mitophagy inducer DFP (Merck, 379–409-5 g) for 24 h and plus-minus a specific inhibitor of vacuolar type H^+^-ATPase activity ConA (Santa Cruz Biotechnology, sc-202111A).

### MS samples preparation

Pellets were lysed in 1% sodium deoxycholate buffer (w/w; Fluka 30,970) in 50 mM ammonium bicarbonate, pH 8.5. Samples were supplemented with 1 unit/µl benzonase (Dr. Nuclease Benzonase; Syd Labs, BP4200).

Protein concentration was measured and adjusted using BCA assay and samples were spiked with 12 or 120 fmol of heavy-labeled peptides. Samples were reduced by adding 1 mM DTT and incubated at 37°C for 30 min, then alkylated with IAA (Fluka, I1149) for 15 min in the dark at room temperature for 15 min.

Samples were digested with trypsin to a 1:100 trypsin (Promega, V5113) to protein ratio for 15 h at 37°C with constant agitation. Trypsin was inhibited and sodium deoxycholate was precipitated adding 50% TFA to a final concentration of 2%. Samples were desalted using AssayMAP C18 cartridges (Agilent, G5496–60033) on a Bravo liquid handling platform (Agilent) and eluted in 50 µl 80% acetonitrile and 0.1% formic acid. Solvents were lyophilized to remove organic solvents. Peptides were resuspended in 0.1% formic acid to a 1 µg/µl.

### Synthetic peptides

Heavy-labeled synthetic standards for all peptides mentioned in Table S1 were acquired from SpikeTides (JPT) or custom synthesis (GenScript) with the following chemical modifications: Carbamoylmethylated cysteine, carboxy-terminal^13^C_6_-^15^N_2_ lysines, ^13^C_6_-^15^N_4_ arginines. Peptides were not purified but isotope label purity was high. Crude peptides were used, and the purity of peptides was not taken into account for calculating concentrations.

### LC-MS/MS

LC-MS/MS measurements were performed on an EASY-nLC 1000 nano-flow UHPLC system (Thermo Fisher Scientific) coupled to a Q Exactive HF-X hybrid quadrupole-Orbitrap mass spectrometer (Thermo Fisher Scientific). Five µl of solubilized peptides in solvent A (0.1% formic acid in water) were separated on a fused silica HPLC column (75 μm internal diameter column Fused-silica PicoTip® emitter: SilicaTip™; New Objective, FS360-75-10-N-5-C25) self-packed with Waters Acquity CSH C18, 1.7 μm (Waters, WAT086529) to a length of 20 cm) using a linear gradient of solvent A and solvent B (0.1% formic acid in 80% acetonitrile in water) from 4% solvent B to 30% over 85 min, followed by an increase to 100% buffer B over 8 min and 7 min at 100% buffer B at a 250 nl/min flow rate. The spray voltage was set to 2.3 kV with a capillary temperature of 250°C.

Mass spectrometer was operated in PRM mode at 60,000 resolution with an AGC target set as 1e6 and a maximum injection time of 118 ms. Isolation window was set at 1.5 m/z, normalized collision energy was set at 27. Data was acquired in centroid mode. Spectral libraries were acquired using a mixture of the synthetic peptides of reference in Data Dependent acquisition with the same resolution and collision energies.

### Data analysis

Raw data was analyzed using Skyline [69,70]. MS2 spectra were matched to libraries generated using the synthetic peptides. Extracted ion chromatograms were manually inspected, signal from interfering ions were removed and integration peak boundaries were modified if needed. Precursors with less than 3 valid transitions were excluded from further considerations. To normalize the intensity across runs, an additional peptide from the background matrix QSVENDIHGLR from type I cytoskeletal KRT18 (keratin 18) was monitored for the calibration curve. For experiments, precursor intensities were normalized on their respective heavy-labeled peptides. Fitting of calibration curves was performed using the MSStats LOBD package [44].

Further data analysis was performed using in-house R code. Peptides intensities were calculated as the sum of light fragments divided by the sum of heavy fragments. Protein intensities were calculated as the average intensities of light fragments with heavy counterparts divided by the heavy fragments intensities for all peptides belonging to this protein if they reached the LOQ in a minimum of a replicate. Heatmaps were generated using the Complex Heatmap R Package [71].

### Flow cytometry

Medium from each well was collected in the appropriate flow cytometry tube. Cells were washed with 500 μL of sterile PBS to remove the remaining culture media and PBS was also collected. Cells were trypsinized adding 150 μL of 0.05% Trypsin (Gibco 25,300–054) per well during 5 min in an incubator at 37°C and 5% CO_2_. To stop the reaction 450 μL of complete culture medium was added and detached cells were immediately collected in the flow cytometry tube. Cells were pelleted by centrifugation, the supernatant was discarded and resuspended in 100 μL of complete medium without phenol red.

Tubes were kept in ice until acquisition. Propidium iodide (Sigma-Aldrich, P1304MP) was added to each tube 5 minutes before being acquired, reaching a final concentration of 100 ng/mL, for viability selection. The analysis was performed in a Cytek Aurora 5 lasers (Cytek Biosciences) acquiring a minimum of 10,000 events per sample. Control cells were used to set the threshold for the mitophagyhigh population defined by a mCherry:GFP ratio of ~ 5%. Unmixing of the data was performed using the Spectroflo (Cytek Biosciences) and the analysis of the unmixed data was conducted in Flowjo v10.10 (BD Biosciences).

### Protein extraction and western blot

Cells were washed with PBS before harvesting by scrapping with lysis buffer (Tris-HCl 50 mM, pH 7.5, glycerol 10%, SDS 2%, protease (cOmplete; Roche 5,056,489,001) and phosphatase (PhosStop; Sigma-Aldrich 4,906,837,001) inhibitors) and boiled for 15 min at 95°C. Protein concentration was determined with the Pierce BCA Protein Assay (ThermoFisher 23,227) following the manufacturer’s instructions. Protein extract (20 µg) was supplemented with 5X loading buffer (4% glycerol, 0.5 M Tris-HCl, pH 6.8, 8% SDS, 0.04% bromophenol blue, 5% β-mercaptoethanol) and resolved on Any kDa Criterion TGX Precast Stain-free gels (Bio-Rad 5,678,124). Proteins were transferred to 0.2 µm PVDF membranes using a TransBlot Turbo Transfer System (Bio-Rad). Membranes were blocked with 5% nonfat milk in PBS-T (0.5% Tween-20, Bio-Rad [1706531] in PBS) for 1 h. Membranes were then incubated overnight at 4°C in primary antibodies diluted 1:1000 in 3% dry milk in PBS-T + 0.02% sodium azide, and subsequently for 1 h at room temperature in secondary antibodies diluted 1:5000 in 3% dry milk in PBS-T. Membranes were developed using Pierce ECL Western Blotting substrate (Thermo Fisher 32,106) and in ECL select western blotting (Cytiva, GERPN2235). The following antibodies were used: anti-CALCOCO2/NDP52 (Atlas, HPA023295), anti-LC3 (Nanotools, 0231–100/LC3-5F10; Novus, NB100–2220), anti-TAX1BP1 (Sigma, HPA02432), anti-SQSTM1 (Cell Signaling Technology, 5114S; Abcam, ab56416), anti-NBR1 (Cell Signaling Technology, 5202S; Santa Cruz Biotechnology, SC-130380), anti-OPTN (Santa Cruz Biotechnology, SC-166576), anti-ACTB/beta actin (Santa Cruz Biotechnology, SC-47778HRP).

### Assessment of mitophagy by fluorescence imaging

Spinning disk confocal microscopy (VisiScope CSU-W1, Visitron System GmbH) was used for *in vivo* imaging. Nuclei were stained with Hoechst and the images were acquired with Photometrics pco.edge 4.2 sCMOS camera, a 40× objective and 0.35 µm z-step. Images with the *mito*-QC reporter to quantify mitophagy were acquired using a Leica SP5 laser scanning confocal microscope (HC PL APO 63×/1.40 oil CS2). All the images were processed with FIJI v1.54f software (ImageJ, NIH). Quantification of mitophagy was performed from five to ten independent fields counting over 200 cells for condition. Images were processed with the *mito*-QC Counter, as described in [72]. For images acquired the following parameters were used: Radius for smoothing images = 1, Ratio threshold = 0.5, and Red channel threshold=mean +0.5 standard deviation.

## Supplementary Material

TableS5.xlsx

TableS4.xlsx

SupplementaryMaterial R4.docx

TableS6.xlsx

## Data Availability

MS raw data and skyline files are available from Panorama (https://panoramaweb.org). R scripts used for data analysis are available from GitHub (https://github.com/DengjelLab/Autophagy-Readout-PRM).
