# Supplementary material for: Targeted proteomics addresses selectivity and complexity of protein degradation by autophagy
Source: Autophagy. 2024 Sep 8;21(2):460–75. doi: 10.1080/15548627.2024.2396792 (PMC11759517; doi:10.1080/15548627.2024.2396792)
Supplement: SupplementaryMaterial R4.docx [file KAUP_A_2396792_SM5155.docx]

**Targeted proteomics addresses selectivity and complexity of protein degradation by autophagy**

Alexandre Leytens^a^, Rocío Benítez-Fernández^b^, Carlos Jiménez-García^b^, Carole Roubaty^a^, Michael Stumpe^a^, Patricia Boya^b^, and Jörn Dengjel^a, *^

^a^Department of Biology, University of Fribourg, Chemin du Musée 10, 1700 Fribourg, Switzerland

^b^Department of Neuroscience and Movement Science, University of Fribourg, Chemin du Musée 14, 1700 Fribourg, Switzerland

**Contact:** Joern Dengjel [joern.dengjel@unifr.ch](mailto:joern.dengjel@unifr.ch) Department of Biology, University of Fribourg, Chemin du Musée 10, 1700 Fribourg, Switzerland

**Supplementary Material**

- **Table S1.** Target proteins monitored by PRM.
- **Table S2.** Validated peptides and their analytical figures of merit.
- **Table S3.** Synthetic PRM peptides from indicated source proteins that are listed in <https://www.phosphosite.org/> as potentially carrying indicated PTMs.
- **Figure S1.** Peptide correlations across experiments.
- **Figure S2.** Extracted ions chromatograms.

**Table S1.** Target proteins monitored by PRM.

| **Protein description** | **UniProt accession** | **Gene name** | **Details** | **Reference** |
| --- | --- | --- | --- | --- |
| microtubule associated protein 1 light chain 3 alpha | Q9H492 | *MAP1LC3A* | Mammalian Atg8 protein | [56] |
| microtubule associated protein 1 light chain 3 gamma | Q9BXW4 | *MAP1LC3C* | Mammalian Atg8 protein | [56] |
| GABA type A receptor-associated protein | Q9GJW7 | *GABARAP* | Mammalian Atg8 protein | [56] |
| autophagy related 4B cysteine peptidase | Q9Y4P1 | *ATG4B* | Lipidation machinery | [56] |
| autophagy related 2A | Q2TAZ0 | *ATG2A* | Lipidation machinery | [56] |
| autophagy related 3 | Q9NT62 | *ATG3* | Lipidation machinery | [56] |
| autophagy related 7 | O95352 | *ATG7* | Lipidation machinery | [56] |
| autophagy related 9A | Q7Z3C6 | *ATG9A* | Autophagosome biogenesis | [1] |
| BCL2 interacting protein 1 | Q12981 | *BNIP1* | Autophagosome biogenesis | [57] |
| GABA type A receptor associated protein like 1 | Q9H0R8 | *GABARAPL1* | Mammalian Atg8 protein | [56] |
| transcription factor EB | P19484 | *TFEB* | Lysosomal biogenesis | [1] |
| WD repeat domain, phosphoinositide-interacting protein 2 | Q9Y4P8 | *WIPI2* | Autophagosome biogenesis | [1] |
| NBR1 autophagy cargo receptor | Q14596 | *NBR1* | Ubiquitinated substrate receptor, pexophagy receptor | [58] |
| sequestosome 1 | Q13501 | *SQSTM1* | Ubiquitylated substrate receptor | [59] |
| autophagy related 12 | O94817 | *ATG12* | Lipidation machinery | [56] |
| calcium binding and coiled-coil domain 2 | Q13137 | *CALCOCO2* | Ubiquitylated substrate receptor | [60] |
| autophagy related 5 | Q9H1Y0 | *ATG5* | Lipidation machinery | [56] |
| autophagy related 4A cysteine peptidase | Q8WYN0 | *ATG4A* | Lipidation machinery | [56] |
| ribosomal protein S6 kinase B1 | P23443 | *RPS6KB1* | Signaling | [61] |
| unc-51 like autophagy activating kinase 1 | O75385 | *ULK1* | Signaling, initiation | [1] |
| RB1 inducible coiled-coil 1 | Q8TDY2 | *RB1CC1* | Autophagy initiation | [1] |
| nuclear FMR1 interacting protein 1 | Q9UHK0 | *NUFIP1* | Proposed ribophagy receptor | [62] |
| starch binding domain 1 | O95210 | *STBD1* | Glycophagy receptor | [63] |
| BCL2 interacting protein 3 like | O60238 | *BNIP3L* | Mitophagy receptor, pexophagy receptor | [64] |
| BCL2 interacting protein 3 | Q12983 | *BNIP3* | Mitophagy receptor | [65] |
| cell cycle progression 1 | Q9ULG6 | *CCPG1* | Reticulophagy receptor | [34] |
| nuclear receptor coactivator 4 | Q13772 | *NCOA4* | Ferritinophagy receptor | [33] |
| toll interacting protein | Q9H0E2 | *TOLLIP* | Ubiquitinated substrate receptor | [32] |
| TNFAIP3 interacting protein 1 | Q15025 | *TNIP1* | Autophagy receptor | [17] |
| Tax1 binding protein 1 | Q86VP1 | *TAX1BP1* | Ubiquitinated substrate receptor | [66] |
| WW domain binding protein 2 | Q969T9 | *WBP2* | Autophagosome biogenesis |  |
| WD repeat and FYVE domain containing 3 | Q8IZQ1 | *WDFY3/Alfy* | Ubiquitylated substrate receptor | [67,68] |
| reticulophagy regulator 1 | Q9H6L5 | *RETREG1/FAM134B* | Reticulophagy receptor | [35] |
| autophagy related 13 | O75143 | *ATG13* | Initiation | [1] |
| reticulon 3 | O95197 | *RTN3* | Reticulophagy receptor | [69] |
| FKBP prolyl isomerase 8 | Q14318 | *FKBP8* | Mitophagy receptor | [30] |
| GABA type A receptor associated protein like 2 | P60520 | *GABARAPL2* | Mammalian Atg8 protein | [56] |
| autophagy and beclin 1 regulator 1 | Q9C0C7 | *AMBRA1* | Mitophagy receptor | [70] |
| [patatin like phospholipase domain containing 2](https://www.genenames.org/data/gene-symbol-report/#!/hgnc_id/HGNC:30802) | Q96AD5 | *PNPLA2* | Lipophagy receptor | [71] |
| prohibitin 2 | Q99623 | *PHB2* | Mitophagy receptor | [31] |
| optineurin | Q96CV9 | *OPTN* | Ubiquitinated substrate receptor | [72] |

**Table S2.** Validated peptides and their analytical figures of merit.

| **Protein accession** | **Protein name** | **Peptide** | **LOD** | **LOQ** |
| --- | --- | --- | --- | --- |
| Q9C0C7 | AMBRA1 | TVGVAFNQETGHWER | 336.2 | 388.5 |
|  |  | WLPEPGLGLAYGTNK | 247.4 | 301.8 |
| O94817 | ATG12 | TIQGLIDFIK | 194.7 | 208.0 |
| O75143 | ATG13 | ASPHDVLETIFVR | 880.1 | 1002.0 |
|  |  | EGGVPLAPNQPVHGTQADQER | 34.2 | 40.9 |
|  |  | SSSSPTGSDWFNLAIK | 80.6 | 104.4 |
| Q2TAZ0 | ATG2A | DLADALLDTER | 8.5 | 15.5 |
|  |  | DLLWLPIEQYR | 251.3 | 297.0 |
|  |  | FTSEVPIWLDYHGK | 72.3 | 81.5 |
| Q9NT62 | ATG3 | ALEVAEYLTPVLK | 193.8 | 205.9 |
|  |  | FVQAVIPTIEYDYTR | 37.5 | 526.1 |
|  |  | TDAGGEDAILQTR | 2.7 | 5.0 |
| Q8WYN0 | ATG4A | LGINQINPVYVDAFK | 460.7 | 474.9 |
|  |  | LLSDISAR | 11.3 | 15.3 |
|  |  | SIGEWFGPNTVAQVLK | 80.5 | 90.3 |
| Q9H1Y0 | ATG5 | EAEPYYLLLPR | 52.5 | 57.9 |
|  |  | IPTCFTLYQDEITER | 348.8 | 378.4 |
|  |  | VSYLTLVTDK | 170.3 | 185.4 |
| O95352 | ATG7 | DVAHSIIFEVK | 208.1 | 230.5 |
|  |  | GYYYNGDSAGLPAR | 8.4 | 13.6 |
| Q7Z3C6 | ATG9A | ALEIIDFFR | 46.0 | 51.8 |
|  |  | LEASYSDSPPGEEDLLVHVAEGSK | 320.7 | 383.6 |
|  |  | SFSPLQPGQAPTGR | 10.4 | 15.8 |
| Q12981 | BNIP1 | AELLQGGDLLR | 4.0 | 7.0 |
|  |  | TILDANEEFK | 47.2 | 58.1 |
| Q12983 | BNIP3 | ASETDTHSIGEK | 102.5 | 121.9 |
|  |  | ILLDAQHESGR | 4.6 | 15.2 |
| O60238 | BNIP3L | DHSSQSEEEVVEGEK | 24.7 | 34.4 |
|  |  | ILLDAQHESGQSSSR | 12.0 | 12.8 |
| Q13137 | CALCOCO2 | DYWETELLQLK | 762.3 | 781.3 |
|  |  | ENDHLFLSLTEQR | 13.8 | 21.4 |
| Q9ULG6 | CCPG1 | FFLNGVFIHDQK | 301.7 | 329.9 |
|  |  | GELQQLSGSQLHGK | 277.5 | 313.9 |
| Q9H6L5 | RETREG1/FAM134B | LDFGIGEYINQK | 60.0 | 71.2 |
|  |  | SLSESWEVINSKPDERPR | 72.9 | 102.2 |
| Q14318 | FKBP8 | ADFVLAANSYDLAIK | 293.6 | 339.6 |
|  |  | VLAQQGEYSEAIPILR | 195.4 | 221.4 |
| Q9H0R8 | GABARAPL1 | EDHPFEYR | 23.1 | 45.3 |
| P60520 | GABARAPL2 | IQLPSEK | 4.6 | 8.4 |
|  |  | VSGSQIVDIDK | 12.9 | 17.7 |
| Q14596 | NBR1 | GALSVAASAYK | 45.0 | 55.3 |
|  |  | IHLWNSIHGLQSPK | 339.7 | 406.3 |
|  |  | VSFDLNTIQIK | 22.8 | 24.7 |
| Q13772 | NCOA4 | DLELAIGGVLR | 28.0 | 47.9 |
| Q9UHK0 | NUFIP1 | DFFGLDTNSAK | 5.1 | 9.6 |
|  |  | DYHNYQTLFEPR | 240.6 | 283.2 |
| Q96CV9 | OPTN | ADLLGIVSELQLK | 2.3 | 15.5 |
|  |  | LNSSGSSEDSFVEIR | 206.3 | 249.4 |
|  |  | TSDSDQQAYLVQR | 122.0 | 143.0 |
| Q99623 | PHB2 | IGGVQQDTILAEGLHFR | 264.0 | 294.4 |
|  |  | IVQAEGEAEAAK | 2.0 | 3.0 |
|  |  | LLLGAGAVAYGVR | 55.4 | 62.4 |
| Q96AD5 | PNPLA2 | VSDGENVIISHFNSK | 196.8 | 267.8 |
|  |  | YVDGGISDNLPLYELK | 45.9 | 54.8 |
| Q8TDY2 | RB1CC1 | ELAQGFLANQK | 0.1 | 1.1 |
|  |  | SLLEQETENLR | 15.0 | 18.0 |
| P23443 | RPS6KB1 | FSPGDFWGR | 35.5 | 46.8 |
|  |  | HINWEELLAR | 287.4 | 337.5 |
|  |  | LGAGPGDAGEVQAHPFFR | 50.2 | 58.8 |
| O95197 | RTN3 | TTPPVEVLHENESGGSEIK | 56.7 | 65.4 |
|  |  | VEGIYTYSLSPSK | 25.6 | 30.6 |
| Q13501 | SQSTM1 | EAALYPHLPPEADPR | 214.8 | 243.9 |
|  |  | LAFPSPFGHLSEGFSHSR | 1227.8 | 1375.8 |
|  |  | LTPVSPESSSTEEK | 3.7 | 6.1 |
|  |  | NYDIGAALDTIQYSK | 203.5 | 238.9 |
|  |  | VAALFPALR | 1.8 | 5.5 |
| O95210 | STBD1 | EHVPSGQFPDTEAPATSETSNSR | 70.3 | 90.3 |
|  |  | FVLVENGGVTR | 72.2 | 88.9 |
|  |  | HSSWGDVGVGGSLK | 11.5 | 34.6 |
| Q86VP1 | TAX1BP1 | AHQLEEDIVSVTHK | 75.6 | 82.6 |
|  |  | LSDQSANNNNVFTK | 20.1 | 20.8 |
| P19484 | TFEB | ANDLDVR | 102.2 | 150.6 |
|  |  | EQAQQEEQR | 0.0 | 917.2 |
|  |  | EYLSETYGNK | 2.1 | 6.9 |
|  |  | FAAHISPAQGSPK | 57.8 | 81.4 |
|  |  | FAAHISPAQGSPKPPPAASPGVR | 94.8 | 109.7 |
| Q15025 | TNIP1 | IYDPGGSVPSGEASAAFER | 33.3 | 37.1 |
|  |  | LQAQVTLSNAQLK | 365.4 | 485.9 |
| Q9H0E2 | TOLLIP | GPVYIGELPQDFLR | 52.9 | 62.4 |
|  |  | IAWTHITIPESLR | 79.1 | 105.7 |
|  |  | LGYAVYETPTAHNGAK | 101.9 | 125.7 |
| O75385 | ULK1 | APFQASSPQDLR | 5.8 | 8.1 |
|  |  | LPDFLQR | 6.4 | 14.8 |
|  |  | TLSEDTIR | 8.8 | 15.8 |
|  |  | TLVPTIPR | 8.5 | 13.1 |
|  |  | TPSSQNLLALLAR | 150.8 | 174.5 |
|  |  | VAELLSSGLQSAIDQIR | 16.2 | 24.1 |
| Q969T9 | WBP2 | AEAGGGWEGSASYK | 59.3 | 73.1 |
|  |  | QPVFGANYIK | 249.9 | 277.6 |
| Q8IZQ1 | WDFY3 | SEGVVPSPVSLVPEEK | 13.7 | 14.3 |
| Q9Y4P8 | WIPI2 | EKPPEEPTTWTGYFGK | 54.5 | 62.0 |
|  |  | FFSLSSVDK | 5.0 | 7.4 |
|  |  | GTYVPSSPTR | 7.1 | 8.5 |
|  |  | VFSIPEGQK | 96.9 | 110.7 |

**Table S3.** Synthetic PRM peptides from indicated source proteins that are listed in <https://www.phosphosite.org/> as potentially carrying indicated PTMs.

| **Protein** | **Peptide** | **Position** | **Modification** | **HTP/LTP** |
| --- | --- | --- | --- | --- |
| OPTN | K.LNSSGSSEDSFVEI**R**.M [168, 182] | 177 | P | 31/9 |
|  | R.TSDSDQQAYLVQ**R**.G [525, 537] | 526 | P | 19/0 |
|  |  | 533 | P | 17/0 |
| ATG3 | K.ALEVAEYLTPVL**K**.E [12, 24] | 18 | P | 329/0 |
| ATG9A | R.LEASYSDSPPGEEDLLVHVAEGS**K**.S [11, 34] | 14 | P | 29/1 |
|  |  | 18 | P | 24/0 |
|  | R.SFSPLQPGQAPTG**R**.A [654, 667] | 656 | P | 23/1 |
| TFEB | K.FAAHISPAQGSP**K**.P [104, 116] | 109 | P | 26/1 |
|  |  | 114 | P | 35/2 |
|  | K.FAAHISPAQGSPKPPPAASPGV**R**.A [104, 126] | 122 | P | 37/5 |
| WIPI2 | K.GTYVPSSPT**R**.L [407, 416] | 412 | P | 12/0 |
|  |  | 413 | P | 30/1 |
|  |  | 415 | P | 11/0 |
| SQSTM1 | K.LAFPSPFGHLSEGFSHS**R**.W [166, 183] | 170 | P | 12/0 |
|  | R.LTPVSPESSSTEEK.S [268, 281] | 269 | P | 208/8 |
|  |  | 272 | P | 355/8 |
|  |  | 275 | P | 35/0 |
|  |  | 276 | P | 37/0 |
|  |  | 277 | P | 44/0 |
|  |  | 278 | P | 12/0 |
|  | K.NYDIGAALDTIQYS**K**.H [421, 435] | 435 | Ubi | 105/1 |
| CALCOCO2 | K.DYWETELLQL**K**.E [203, 213] | 204 | P | 15/0 |
| RPS6KB1 | K.FSPGDFWG**R**.G [451, 459] | 452 | P | 98/2 |
| ULK1 | K.TPSSQNLLALLA**R**.Q [636, 648] | 638 | P | 75/4 |
|  |  | 639 | P | 12/2 |
| STBD1 | R.HSSWGDVGVGGSL**K**.A [209, 222] | 211 | P | 17/1 |
| BNIP3L | R.DHSSQSEEEVVEGE**K**.E [115, 129] | 117 | P | 21/0 |
|  |  | 118 | P | 23/0 |
|  |  | 120 | P | 35/0 |
| BNIP3 | R.ASETDTHSIGE**K**.N [79, 90] | 86 | P | 30/0 |
| TOLLIP | R.LGYAVYETPTAHNGA**K**.N [81, 96] | 96 | Ubi | 20/0 |
|  | R.IAWTHITIPESL**R**.Q [131, 143] | 143 | Ubi | 31/0 |
| ATG13 | R.ASPHDVLETIFV**R**.K [360, 372] | 361 | P | 23/0 |
| RTN3 | K.VEGIYTYSLSPS**K**.V [220, 232] | 224 | P | 22/0 |
| FKBP8 | K.VLAQQGEYSEAIPIL**R**.A [315, 330] | 322 | P | 150/0 |

Data were filtered on a minimum of 10 identifications in high throughput studies (HTP/LTP column). Numbers in square brackets indicate start and end amino acid position. “Position” indicates position of PTM. P: phosphorylation; Ubi, ubiquitination; HTP: high-throughput study; LTP: low-throughput study.


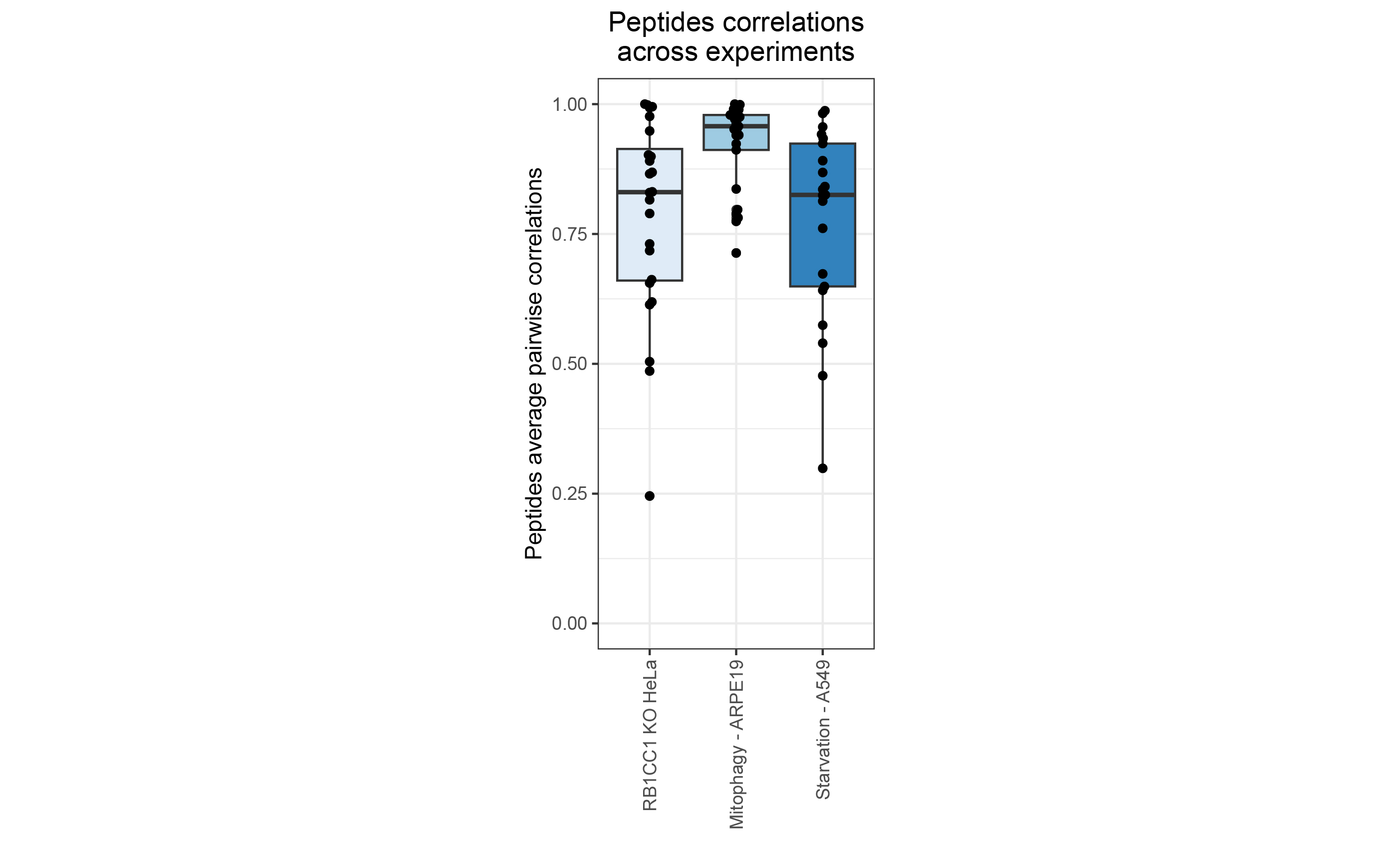


**Figure S1.** Peptide correlations across experiments. Points represent the average pairwise correlations across the entire experiment for peptides originating from the same source protein, for all proteins quantified with more than a single peptide. Medians of peptide correlations are: *RB1CC1* KO HeLa: 0.830; Mitophagy - ARPE19: 0.955; Starvation - A549: 0.825.

**
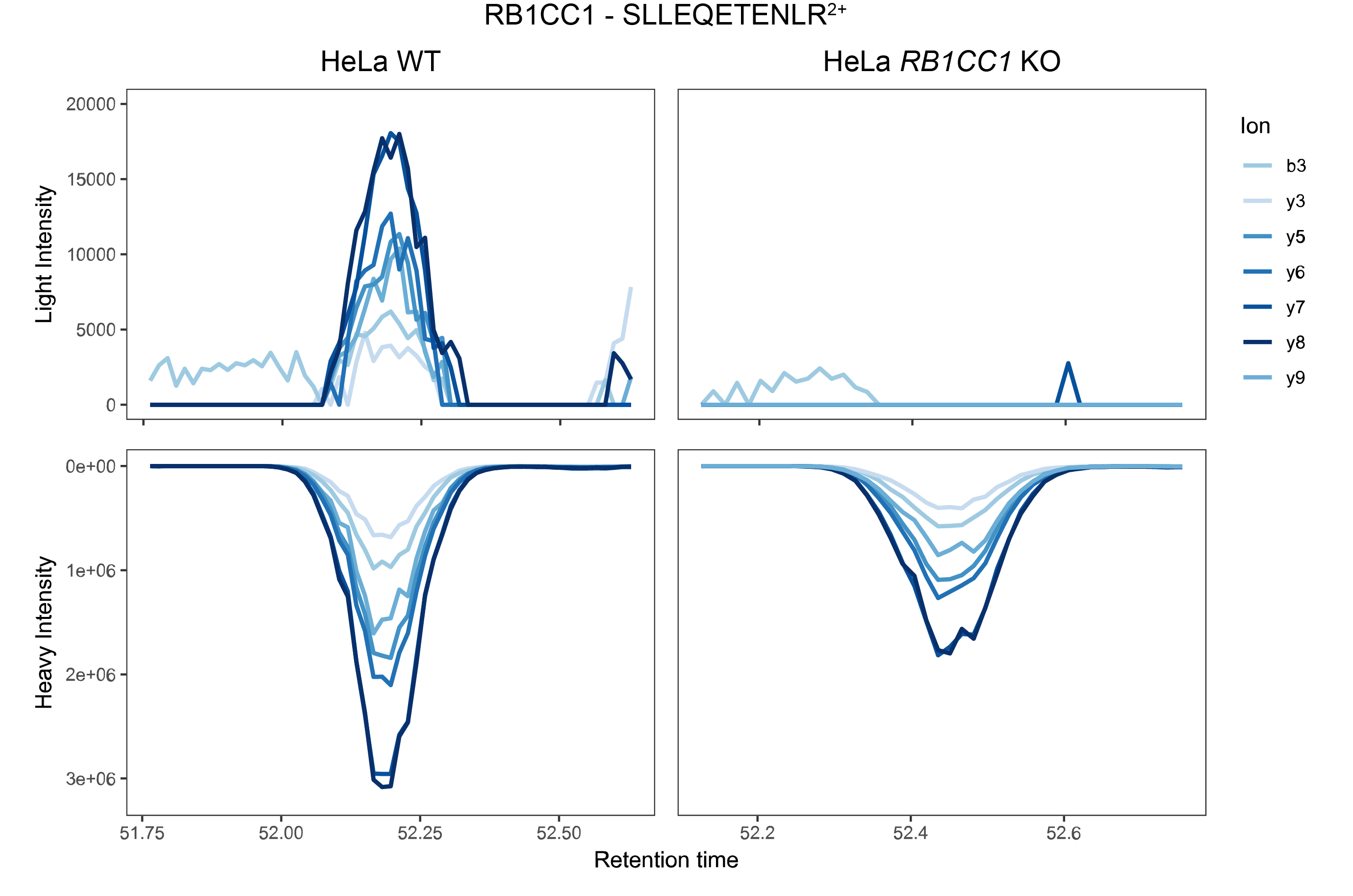
**

**Figure S2.** Extracted ions chromatograms (XICs). The XICs of indicated fragments of the doubly charged peptide SLLEQETENLR (RB1CC1/FIP200[1091-1101]), one of the peptides used as proxy to measure RB1CC1 abundance, are shown. Upper panels represent the light signals from endogenous peptides, lower panels represent the heavy signals from heavy-labelled spiked-in reference peptides.
